# Understanding the palliative care needs and experiences of people with mesothelioma and their family carers: An integrative systematic review

**DOI:** 10.1177/02692163211007379

**Published:** 2021-04-08

**Authors:** Madeleine Harrison, Clare Gardiner, Bethany Taylor, Stephanie Ejegi-Memeh, Liz Darlison

**Affiliations:** 1 Division of Nursing and Midwifery, Health Sciences School, University of Sheffield, Sheffield, UK; 2 The Glenfield Hospital, University Hospitals of Leicester NHS Trust, Leicester, UK; 3 Mesothelioma UK, Leicester, UK

**Keywords:** Mesothelioma, palliative care, terminal care, family caregivers, review

## Abstract

**Background:**

People with mesothelioma and their families have palliative care needs throughout the relatively short trajectory of their illness.

**Aim:**

To describe the palliative care needs and experiences of people with mesothelioma and their family carers.

**Design:**

Integrative systematic review with narrative synthesis (PROSPERO: CRD42020190115).

**Data sources:**

MEDLINE, CINAHL, PsycINFO and the Cochrane Library were searched for articles published between 01 January 2000 and 10 May 2020. Articles were included if they presented empirical studies or comprehensive reviews including information about the palliative care needs and experiences of people with mesothelioma and their family carers.

**Results:**

The search yielded 508 articles, 14 were included in the analysis. A cross cutting theme of ‘uncertainty’ was identified encompassing five themes: (1) organisation and co-ordination of services, (2) communication and information needs, (3) management of care needs and high symptom burden, (4) consideration of the impact of seeking compensation and (5) family carer needs. Our findings demonstrate that people with mesothelioma want a co-ordinated, team-based approach to palliative care with a named point of contact. Whilst carers value and benefit from early referral to specialist palliative care, this does not necessarily reflect the outcomes and views of patients.

**Conclusion:**

The evidence base around the palliative care needs and experiences of people with mesothelioma and their carers needs to be strengthened. The results of this review support the need to develop a greater understanding about the role non-specialist palliative care clinicians’ play in providing generalist palliative care for people with mesothelioma and their carers.

**What is already known about the topic?**

- Mesothelioma is a rare cancer associated with a high symptom burden and limited survival, typically 12 months.
- The pain and dyspnea associated with mesothelioma are perceived to be difficult to palliate.
- Early referral to specialist palliative care was not found to improve quality of life or mood for patients with mesothelioma when compared with standard care alone, which could be interpreted to mean that standard care is sufficient.

**What this paper adds?**

- People with mesothelioma live with unfathomable uncertainty resulting from the lack of clarity around disease progression and when death will occur, and consequently expressed a need for clearer information and an accessible contact person with oversight of their care.
- A co-ordinated, team-based approach to palliative care was preferred, however the majority of the mesothelioma literature only explicitly recognised palliative care provision when it was provided by specialist palliative care teams and did not distinguish between the different levels of palliative care services (i.e. generalist and specialist palliative care).
- The process of seeking compensation compounds an already difficult situation by dictating how the limited time the patient and carer have left together is spent.

**Implications for practice, theory or policy**

- Greater consideration of the role non-specialist palliative care clinicians’ play in providing generalist palliative care in mesothelioma is required, as well as increased partnership working with specialist palliative care.
- The unique circumstances regarding compensation in asbestos related disease should be considered an important element of palliative care for this group.

## Introduction

Mesothelioma is a rare, incurable cancer with a high symptom burden.^1^ Predominantly a pleural disease affecting the lining of the lungs, it can also occur in the peritoneum.^2^ The incidence of mesothelioma varies within and between countries, with the highest rates seen in Australia and the United Kingdom (29 cases per million population),^3^ and the number of deaths estimated to be 38,400 per year globally.^4^ Most cases of the disease are caused by preventable asbestos exposure, which usually occurs in the workplace resulting in mesothelioma being classified as an industrial disease.^5,6^

People with mesothelioma experience a range of debilitating symptoms including pain, breathlessness, cough, fatigue, sweating and weight loss, as well as anxiety and low mood.^7^ There are currently no curative treatments for mesothelioma, but medicine, surgery and radiotherapy can help to improve survival and palliate symptoms.^8^ The main source of support for people with mesothelioma are their family and friends.^9^ Family carers bear a significant emotional and physical burden when caring for people with mesothelioma.^10^

Mesothelioma typically progresses rapidly and the majority of people with the disease will die within a year of diagnosis.^11^ Consequently, people with mesothelioma and their families have palliative care needs throughout the relatively short trajectory of their illness, from diagnosis to the end of life.^12^ There is a perception that the pain and breathlessness experienced by people with mesothelioma are sometimes difficult to palliate.^13^ The British Thoracic Society and the European Respiratory Society highlight the importance of palliative care provision to manage the symptoms of mesothelioma and offer emotional, psychological and spiritual support.^14,15^

There is evidence that specialist palliative care improves the quality of life of patients with palliative care needs arising from a wide range of different life limiting conditions, and at the same time reduces health system costs and resource utilisation.^16^ However, a 2019 multicentre randomised controlled trial in the UK and Australia has shown that early routine specialist palliative care for patients recently diagnosed with malignant pleural mesothelioma does not have any additional impacts on quality of life over standard care.^17^ In this trial, early specialist palliative care appears to have been trialled with people with mesothelioma in response to the finding that the intervention was beneficial in similar populations.^16^ However, there is limited discussion of what the palliative care needs of people with mesothelioma might be, and how these may differ from patients with other malignancies.^17^ Healthcare needs are a complex concept commonly defined as the capacity to benefit.^18^ Where the healthcare needs of a patient population are determined using a top-down approach, which relies too heavily on what a few people perceive to be the needs of that population rather than what they actually are we risk providing ineffective and costly intervention, as demonstrated by this trial.^17,18^ Consequently the authors of this study propose that we need a better understanding of the palliative care needs and experiences of people with mesothelioma and their family carers in order to inform decisions on what services need to be in place, who should provide them and at what time they should be offered. Compared with other cancers, the palliative care needs and experiences of people with mesothelioma pose unique complexities due to the rarity of the disease, the rapidity of decline^19^ and the industrial nature of the disease, which is associated with the need for a legal inquiry by a coroner and protracted compensation claims processes.^20^ The aim of this systematic integrative review was to identify and synthesise existing evidence on the palliative care needs and experiences of people with mesothelioma and their family carers and describe how their needs are being addressed.

## Methods

### Literature review question

The specific question to be addressed was:

What are the palliative care needs and experiences of people with mesothelioma and their family carers?

### Design

An integrative systematic review methodology was employed to enable the inclusion of findings from a diverse range of methodologies (i.e. qualitative and quantitative) in this under researched topic.^21^ The review followed the procedures outlined in the Centre for Reviews and Dissemination (CRD) guidance.^22^ The review protocol is registered in PROSPERO: CRD42020190115. The Preferred Reporting Items for Systematic Reviews and Meta-Analyses (PRISMA) was used as the reporting guideline.^23^

### Search strategy

Electronic searches were undertaken using the following databases: MEDLINE, CINAHL, PsycINFO and the Cochrane Library. Core search terms included: mesothelioma and palliative care or end of life care or terminal care or supportive care or hospice. The search was limited to include papers published from 01 January 2000 to 10 May 2020. The terms were adapted for each database and MeSH terms were used, where possible. Search histories are provided in Supplemental Appendix 1. The reference lists of included papers were searched for potentially relevant articles.

Once duplicates had been removed, the title and abstract of all identified records were screened according to the inclusion and exclusion criteria shown in Table 1 by one reviewer (MH); whilst a second reviewer (CG) independently screened 10% of records to ensure the inclusion and exclusion criteria were being applied with good agreement. Full-text review was carried by two independent reviewers (MH and one of CG/SE/BT). Decisions were documented in separate databases and disagreements were resolved by consensus.

**Table 1.** Inclusion and exclusion criteria.

Inclusion criteria	Exclusion criteria
Empirical research studies (any research design) or comprehensive reviews including information about the palliative care needs* and experiences of people with mesothelioma and their family carers	Not focused on people with mesothelioma/data from people with mesothelioma not presented separately
	No reference to the palliative care needs* or experiences of patients and families
	Conference proceedings/discussion articles/commentary/letters/book chapters without a comprehensive literature review
	Published in a language other than English

* Palliative care need was defined as the capacity to benefit from palliative care.

### Data extraction, analysis and synthesis

The process of extracting and synthesising the data followed the integrative method described by Whittemore and Knafl,^21^ which allows for the synthesis of findings from a diverse range of designs (qualitative, quantitative and reviews). Data reduction and extraction were conducted by one reviewer (MH) using a pre-defined pro forma, including the following: author(s); year of publication; country where study was conducted; study design; population; setting; sample; characteristics of included patients (age, sex, time since diagnosis); patients’ palliative care needs and who identified them; family carers’ palliative care needs and who identified them and how palliative care needs were being addressed. The process of data reduction facilitated the integration of findings from a variety of different study designs in which patients’ and carers’ palliative care needs were measured and reported in different manners.^21^ Data from the pro forma were displayed using a coding matrix in Excel to enable comparison on specific issues or sample characteristics. Two authors (MH and CG) collaboratively reviewed the extracted data noting patterns and themes and drawing conclusions through discussion.^21^

### Quality appraisal

All studies included in the analysis were assessed for methodological quality. Each assessment was carried out by two independent reviewers (MH and one of CG/SE/BT). Where disagreements arose they were discussed and resolved through consensus. The Mixed Methods Appraisal Tool (MMAT) was used to appraise primary research.^24^ The MMAT can be used to assess the methodological quality of five different categories of study design. The tool includes two screening questions to determine whether the study is empirical and five quality criteria for each category of study. To appraise reviews, the Joanna Briggs Institute (JBI) Critical Appraisal Checklist for Systematic Reviews and Research Syntheses was used.^25^ The review checklist includes 11 quality criteria. It is not recommended to present an overall score when using the MMAT,^24^ and since scoring of quality appraisal has been criticised more widely,^26^ a score will not be presented for the JBI review checklist either. The appraisal will instead inform a narrative description of study quality and inform the weight placed on the findings of different studies in the data synthesis. As methodological quality was not established as an inclusion or exclusion criteria, all articles appraised were included in the review.

## Results

**Figure 1.**
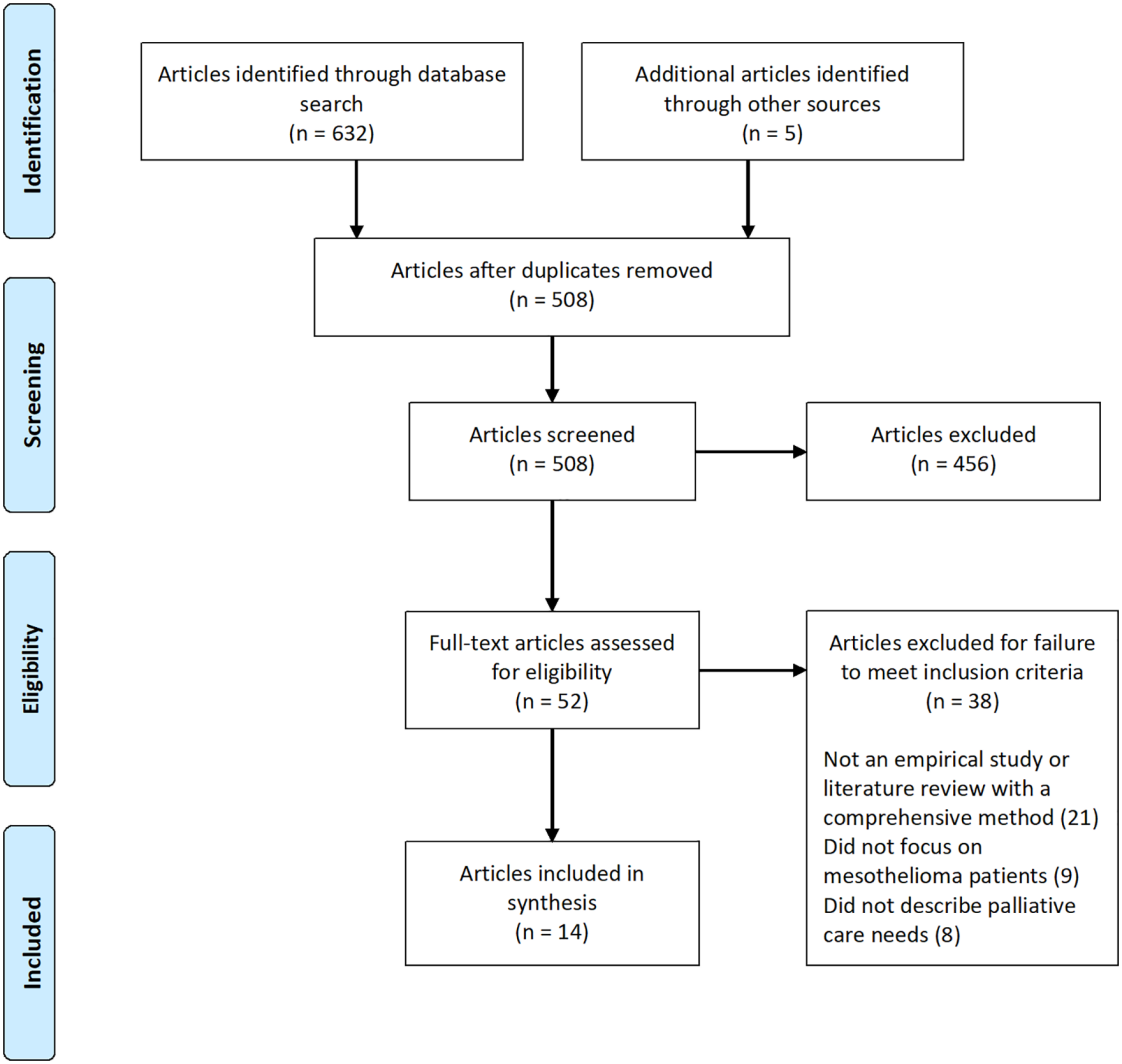
PRISMA flow diagram of study selection.

A total of 508 articles were initially retrieved and 14 met the inclusion criteria. The PRISMA flow diagram in Figure 1 illustrates the article selection process and reasons for exclusion.

### Study characteristics

Table 2 shows a summary of the characteristics of the included studies along with a summary of the main findings relevant to this review. The included studies used a variety of different methodologies: quantitative (*n* = 8), qualitative (*n* = 5) and review (*n* = 1). The majority of studies were conducted in the UK (*n* = 6) or Australia (*n* = 5), with others from Japan (*n* = 2), Italy (*n* = 1) and the United States (*n* = 1). Sample sizes ranged from 2 patients in one of the qualitative studies^27^ to 174 patient participants in a multi-centre randomised controlled trial.^17^ Most articles exclusively included people with mesothelioma (*n* = 11) and their carers (*n* = 5), the others included: nursing staff,^28^ mesothelioma and asbestos related lung cancer (but all patient participants had mesothelioma),^27^ and a review that compared the psychological care needs of patients with mesothelioma and advanced lung cancer.^29^ The majority of articles were limited to people with malignant pleural mesothelioma (*n* = 10), whilst the others were open to people with all types of mesothelioma (*n* = 3). All of the studies that reported the gender of the participants included more males than females, as would be expected given the higher prevalence of mesothelioma in men. Where reported, the average age of patient participants ranged from 51^30^ to 73 years.^31^

**Table 2.** Summarised results of the studies assessed in this systematic review.

First author, country and year	Study aim	Study design and methodology	Population, setting and sample size	Main findings
Arber and Spencer^32^ UK, 2013	To explore the patient’s experience during the first 3 months following a diagnosis of malignant pleural mesothelioma (MPM)	Qualitative	MPM patients recruited from two hospitals	The key concept was ‘uncertainty and lack of control’, which was underpinned by three themes: it’s all bad news, good days and bad days and strategies of amelioration.
		Grounded theory	*N* = 10	Participants described worries about long-term outcome and speed of deterioration even in the early stage after diagnosis. One participant described seeking referral to palliative care. Palliative care referral was a positive experience/turning point. Patients’ lack of control was added to by not understanding who was in charge of their care.
Ball et al.^29^ UK, 2016	To establish whether the psychological needs of patients with pleural mesothelioma are the same as patients with advanced lung cancer	Review	17 articles, including five with MPM patients only and nine with advanced lung cancer only and three mixed MPM and lung cancer	Common themes across the studies were grouped into 10 key concepts: these were uncertainty, normality, hope/hopelessness, stigma/blame/guilt, family/carer concern, physical symptoms, experience of diagnosis, iatrogenic distress, financial/legal and death and dying.
		Meta ethnography	*N* = unknown	Lack of referral to specialist palliative care and supportive care provision was noted as a particular issue for those with MPM. Those with MPM expressed concern about death and the process of dying. As well as the need to ensure their affairs were in order to reduce the burden on family.
Bibby et al.^33^ UK, 2017	To describe the characteristics of patients who chose active symptom control over chemotherapy and explore their reasons for doing so	Quantitative	MPM patients recruited from one hospital	Active symptom control was chosen by 33% of participants. Participants choosing active symptom control were older, had poorer performance status and were more often female. Reasons for choosing active symptom control included: benefits of chemotherapy did not justify the risk of side effects (5/15), a desire to prioritise quality of life in the context of no current symptoms (4/15), needle/hospital phobia (3/15), prior negative experiences of chemotherapy (2/15), pursuit of experimental treatment in another country (1/15). Reasons were only documented for one third of participants who chose active symptom control.
		Prospective observational study	*N* = 139	
Brims et al.^17^ Australia and UK, 2019	To examine the effect of regular early specialist palliative care on HRQoL in patients with MPM and their carers compared with standard care alone	Quantitative	MPM patients and their carers recruited from 19 UK hospitals and one large Australian site	Routine early referral to specialist palliative care did not result in a statistically significant difference in quality of life or mood at 12 and 24 weeks post-randomisation for patients with a good performance status when compared to standard care alone. Carer satisfaction with end-of life care was measured using the FAMCARE-2 questionnaire. Scores were significantly higher in the intervention arm at 12 and 24 weeks indicating that routine early referral to specialist palliative care was helping to meet the needs of carers compared with standard care.
		Randomised controlled trial	*N* = 174 patients	
			*N* = 145 carers	
Clayson et al.^34^ UK, 2005	To describe the experience of mesothelioma and its meaning for patients	Qualitative	MPM patients recruited from three hospitals	The findings were described across four themes: coping with symptoms, the burden of medical interventions, finding out about mesothelioma and psychosocial issues. The offer of palliative care from a Macmillan nurse at the time of diagnosis was distressing and provoked fear. Symptom burden provided a constant reminder of disease progression. Some patients demonstrated acceptance of the terminal nature of the diagnosis, others made plans to deal with their death and possessions. The benefit and compensation process aggravated the situation and persists for relatives after the patients die.
		Grounded theory	*N* = 15	
Dooley et al.^30^ Australia, 2010	To investigate the specific psychological consequences of mesothelioma	Quantitative	Mesothelioma patients recruited via a lawsuit filed against their employer	People with mesothelioma reported higher levels of depression and anxiety and significantly more traumatic stress symptoms than the normative group for the Trauma Symptom Inventory. The symptom cluster related to the re-experiencing domain suggests that the physical manifestation of the illness served as a constant reminder of health status. Clinical interviews highlighted the financial pressure that their illness had placed on their family.
		Retrospective observational study	*N* = 49	
Hughes and Arber^35^ UK, 2008	To explore patients’ lived experience of mesothelioma	Qualitative	MPM patients recruited from local palliative nursing service (Macmillan) referrals	Six themes were identified: physical effects, loss of intimacy, isolation, pursuing compensation, sharing experiences and supportive care. The majority of supportive care was sought out by patients from GPs, specialist palliative care or psychological services. Participants wanted the right support at the right time and suggested that they could not take in information about supportive/palliative care in the early stages due to the overwhelming amount of information provided. Pursuing compensation dictates how limited time together is spent and requires a joint effort between the patient and carer.
		Phenomenological approach	*N* = 5	Carer’s need the opportunity to speak with healthcare professionals in confidence, alone.
Kao et al.^31^ Australia, 2013	To determine the proportion of MPM patients who received active anticancer treatments in the last month of life and identify potential factors associated with chemotherapy use	Quantitative	MPM patients recruited from compensation scheme (Dust Diseases Board of New South Wales)	Patients who received two or more lines of chemotherapy were more likely to be receiving chemotherapy in their last month of life. Patients who received chemotherapy at the end of life had shorter survival compared to those who did not receive chemotherapy at the end of life. Authors suggest careful consideration of when to cease chemotherapy is required, including timely and frank discussion with the patient and their family. There was a trend for lack of palliative care involvement to be associated with use of chemotherapy in the last month of life.
		Retrospective observational study	*N* = 147	
Lee et al.^36^ Australia, 2009	To describe the needs and experiences of people with mesothelioma and asbestos-related lung cancer, their carers and service providers in the Latrobe Valley community	Qualitative	Patients with mesothelioma and asbestos related lung cancer, carers and healthcare/legal professionals recruited from advertisements	Three main themes: illness experience, carer and family roles and services and service gaps. Referral to palliative care occurred late in the illness due to discomfort associated with acknowledgement of dying, which resulted in poor symptom control and a lack of support for carers. A lack of holistic and co-ordinated care was described, as well as difficulty accessing reliable and accurate information. The effort and time required to seek compensation was particularly burdensome given the declining health of the patients.
		Descriptive case study	Mesothelioma patients *N* = 2	Carers expressed concerns about the lack of bereavement services. One carer described palliative care nurses as ‘the angels of death’.
			Carers *N* = 6	
			Professionals *N* = 5	
Mercadante et al.^37^ Italy, 2006	To examine the epidemiological characteristics and symptom burden of mesothelioma patients when admitted to home palliative care	Quantitative	Mesothelioma patients recruited from a home palliative care service	Patients had a consistently high physical and psychological symptom burden (depression and anxiety). Three quarters of patients had pain (18 moderate, 2 severe) despite receiving high doses of opioids. The principal pain site was the chest. Pain was significantly associated with the amount of oral morphine delivered and dyspnea. The average duration of home palliative care admission was 54.8 days (which corresponded with death for all except two participants). Authors thought pain management could have been improved if patients were referred to palliative care earlier.
		Retrospective observational study	*N* = 56	
Nagamatsu et al.^28^ Japan, 2014	To evaluate the effect of both the Educational Program on Palliative Care for Patients with MPM and the Palliative Care for MPM Handbook for Nurses in Japan	Quantitative	Nursing staff recruited nationwide from hospitals, home visiting nurse stations and health care centres	The educational program on palliative care for MPM for nurses was effective in reducing perceived difficulties experienced by nurses caring for patients with MPM. Post-test difficulty scores were lower than the pre-test scores. Participants positively evaluated the educational program for validity, clarity, clinical usefulness and the facilitators. Quotes from the evaluation provided information about the educational needs of staff to enable them to meet the palliative care needs of mesothelioma patients: physicians lack of knowledge about what medicines to prescribe; the lack of information/handbooks on the topic; the desire to learn more about communication with a dying patient; concern about ‘failure’ to control symptoms.
		Prospective pre-test post-test	*N* = 27	
Nagamatsu et al.^38^ Japan, 2019	To determine the needs of patients within the health service by quantifying the requests to their physicians and qualitatively analysing their responses	Quantitative	MPM patients recruited nationwide from cancer hospitals and support groups	Patients with MPM had a variety of unmet needs from their physicians. Patients wanted clear and understandable explanations about MPM, particularly in relation to the curability and prognosis of the disease. Participants wanted their physician to deliver treatment based on the patient’s perspective by accepting and empathising with their anxiety and pain. Physicians conveying information about the benefits of palliative care and advising the patient to introduce it at an early stage was perceived to be helpful as it gave the patient time to prepare. Patients who did not receive palliative care made more physician requests than those who received palliative care.
		Retrospective survey	*N* = 73	
Walker et al.^39^ United States, 2019	To explore the lived experience of MPM in the United States and identify unmet patient needs	Qualitative	MPM patients recruited from University Medical Centre	Three major themes: uncertainty/worry about the future, value in relationships and adapting to a new norm. Uncertainty/worry about the future stemmed from a lack of clarity about when the MPM would progress and how or when death would occur. Awareness of the incurable nature of the condition was referred to as a ‘death sentence’, and the most common coping strategy was ‘taking life one day at a time’. Some employed practical strategies by making advanced preparations. Patients expressed concern about symptom management at the end of life, particularly in relation to pain and breathlessness. MPM patients preferred a personalised, coordinated, team based approach with open and honest communication. Worries about loss of control at the end of life were expressed. Spiritual or religious rituals and support helped participants to maintain a sense of control.
		Phenomenological approach	*N* = 7	
Warby et al.^9^ Australia, 2019	To document the experience of MPM patients and their caregivers	Quantitative	MPM patients and their informal carers recruited from support groups nationwide, a regional compensation scheme and advertisements in two hospitals	A palliative care referral had been received by 31% of patients, compared to 85% of caregivers (many were bereaved). Of the patients who had not received palliative care, none felt it could have helped them, but 13% of carers whose patients did not receive palliative care felt it could have helped them. The majority of participants received ‘sufficient’ support (71%). Thirty-five percent of patients would have liked more information about what to expect with their disease. Almost all patients sought compensation for their MPM (97%).
		Retrospective survey	Patients *N* = 78	Caregivers would have liked to talk to someone by themselves (41%), more time with doctors (30%), access to psychological support (29%) and clearer information (31%). Bereaved caregivers requested grief counselling (39%) and a post-death consultation with a medical (25%) or palliative care specialist (23%).
			Carers *N* = 106	

### Methodological quality

Overall, the methodological quality of the included studies was moderate to high (see Supplemental Appendices 2 and 3). The qualitative studies included were generally of high methodological quality, but there was a lack of information about sampling strategies and little justification of sample size,^32,34,35,36,39^ which ranged from 2^27^ to 15^34^ participants with mesothelioma. The quantitative descriptive studies sometimes failed to ensure the sample was representative of the target population due to recruitment through compensation claims^9,31^ and lawsuits.^30^ However, studies recruiting through clinical caseloads had small sample sizes due to the rarity of the condition.^37^ Response rates across the descriptive studies were relatively low increasing the chance of non-response bias.^9,31,38^ The only randomised controlled trial was methodologically sound, but it was not possible to blind participants or the research team to group allocation due to the nature of the palliative care intervention.^17^ One of the quantitative non-randomised studies was methodologically sound,^33^ but the other attempted to evaluate effectiveness without a control group and did not account for any confounding factors.^28^ The only systematic review to be included in the review lacked a clear search strategy.^29^

### Main findings and key themes

The synthesis highlighted one cross cutting theme of ‘uncertainty’ and five themes within this: (1) organisation and co-ordination of services, (2) communication and information needs, (3) management of care needs and high symptom burden, (4) consideration of the impact of seeking compensation and (5) family carer needs. Each of the five themes will be described in turn.

### Organisation and co-ordination of services

The cross-cutting theme of uncertainty was described in relation to the diagnosis, prognosis and progression of mesothelioma across a number of qualitative studies,^32,34,39^ and a qualitative review.^29^ This uncertainty and loss of control was compounded when patients also did not know who was co-ordinating their care. Care was described as fragmented and uncoordinated,^29^ with patients ‘bewildered’ by various doctors^34^ and patients left feeling that they were navigating the system on their own.^27^ In addition, patients not receiving active anticancer therapy reported feeling abandoned by the system.^35^ Communication between hospital and community services was criticised in qualitative studies^27,35^ and a survey found communication between healthcare professionals was perceived to be variable, with 25% of patients stating that their health professionals talked to each other occasionally or hardly ever.^9^ Studies have highlighted the need for people with mesothelioma to receive a co-ordinated, team-based approach to palliative care, whilst recognising that they need a named point of contact to provide some certainty about who is in charge of their care.^29,32,34,39^

When describing the organisation of palliative care services for people with mesothelioma, studies often lacked clarity in terms of what they meant by palliative care, with few studies making a clear distinction between generalist and specialist palliative care.^17^ However, the language used, such as ‘referral’ or ‘access’ to palliative care,^9,27,31,32,35,37^ demonstrate an underlying assumption, from authors and patient participants, that palliative care is provided by a separate, specialist team rather than by existing members of the patients clinical team. A number of articles made recommendations about the availability of palliative care suggesting early,^31,32,37^ timely^27,34,35^ or more equitable^9^ access is needed. Routine early referral of patients with mesothelioma to specialist palliative care was evaluated in a randomised controlled trial, which found that it was not effective at improving quality of life or mood in patients with good performance status compared with standard care.^17^ Other studies recommending early referral to (specialist) palliative care provided this suggestion on the basis of qualitative reports of patients having to independently seek out a palliative care referral^32,35^ and poor pain management prior to referal.^37^ Additionally, lack of palliative care input was found to be associated with patients expressing more suggestions for improvements in their medical care^38^ and increased likelihood of receiving chemotherapy in the last month of life (which represents poor oncology practice).^31^ However, it is important to note that in this review the need for early referral was not expressed directly by patients or clearly indicated by the findings. A recent survey conducted in Australia found that, of the patients who had not received palliative care, none felt that it could have helped them, whereas 13% of carers thought it would have been helpful.^9^ Offering a palliative care referral at the time of diagnosis when patients felt overwhelmed with new information was perceived to be distressing in two qualitative studies.^34,35^

In contrast, a more recent qualitative study shifts the emphasis from specialist palliative care referral to the role that nursing staff can play in providing supportive care (a term often used synonymously with palliative care).^39^ Nurses are recommended to support patients with malignant pleural mesothelioma to find meaning in their lives and encourage strategies for adapting to a ‘new norm’, as well as promoting control through healthy lifestyle choices, autonomous decision making and spiritual practices.^39^ This is consistent with a suggestion from another study that supportive care could be provided via an early opportunity for a home visit with a clinical nurse specialist or specialist palliative care nurse.^32^ Where patients described the palliative care they had received, it was viewed as beneficial as it provided an opportunity to discuss their anxieties and help them to feel more prepared.^32,35,38^

### Communication and information needs

Most of the articles included in the review (*n* = 9) described difficulties in accessing information to enable people with mesothelioma to make informed treatment decisions and/or poor communication practices between healthcare professionals and patients.^27–29,31,32,35,38,39^ The information and communication needs highlighted were described in relation to patients whole care experience, including palliative care, but not necessarily specifically about palliative care. Studies highlighted difficulties accessing reliable and accurate information,^27^ and clear, understandable explanations.^38,39^ Patients also wanted to have an opportunity to express their illness story and valued the opportunity to be listened to by a healthcare professional; demonstrating the importance of two-way communication.^32,35^ Two studies highlighted the importance of developing relationships with other people with mesothelioma and a need to share their experiences with others in a similar position, where this was not available.^35,39^

The need for open and frank communication when conveying information about the curability, prognosis and progression of the disease was highlighted across multiple studies.^9,31,38,39^ In one survey, 35% of patients would have liked more information about what to expect from their disease, whilst 58% of carers would have liked more information about what to expect in caring for someone with malignant pleural mesothelioma.^9^ A survey in Japan, found that whilst most participants (*n* = 17) wanted clear and complete information about their disease and its prognosis, a smaller number (*n* = 5) wanted the information to be delivered in a more indirect or vague manor.^38^ These differences highlight the importance of a personalised approach to communication and information provision, which was also recommended in a recent qualitative study.^39^ In the same study, patients highlighted that they would like more information about how and when death might occur.^39^ The lack of clear information exacerbated feelings of uncertainty.^29,39^

Information provision was perceived to be the role of healthcare professionals with one article describing the importance of staff being knowledgeable about mesothelioma and dedicated to its treatment.^38^ Walker et al.^39^ emphasise the key role that nursing staff play in communication by assessing information needs and providing personalised education. One article described an evaluation of an educational program on palliative care for patients with malignant pleural mesothelioma for nurses in Japan.^28^ Nurse participants were highly satisfied with the program and its handbook and the evaluation suggested that the training reduced perceived difficulties experienced by nurses caring for people with mesothelioma. Quotes highlighted a desire to learn more about communicating with dying patients; lack of resources about how to provide palliative care for people with mesothelioma and concerns around health care professional’s knowledge of how to control symptoms and what medicines to prescribe.^28^

### Management of care needs and high symptom burden

The high symptom burden experienced by people with mesothelioma was widely described across the included articles.^17,27,28,30,32,33–35,37–40^ Mercadante and colleagues observed that patients had a consistently high physical and psychological symptom burden with patients scoring a mean sum of 37.7/100 on the Edmonton Symptom Assessment System at admission to home palliative care.^37^ A high prevalence of pain, weakness, poor appetite, poor well-being and dyspnea were noted.^37^ Three quarters of patients had pain (18 moderate, 2 severe) on admission despite having received high doses of opioids; pain was found to be associated with the amount of oral morphine delivered and dyspnea.^37^ Patients earlier in the disease trajectory expressed fears of uncontrolled pain,^39^ shortness of breath/suffocation^34,39^ and the process of dying,^29^ whereas those interviewed later in the trajectory redefined the meaning of their symptoms as the disease developed.^34^ The heavy symptom burden provided a constant reminder of disease progression and health status.^30,34^ The poor prognosis related to mesothelioma caused a dilemma for patients about whether to initiate and/or continue treatment given the limited gains offered and the considerable side effects associated with treatment.^32,33^ An observational study conducted in a single UK centre found 33% of people with mesothelioma chose active symptom control; the primary reasons for declining anti-cancer treatment were concerns over side effects, the modest survival benefit and previous negative chemotherapy experiences.^33^ Those choosing active symptom control were older (mean age 74 vs 68), had a poorer performance status and were more often female (24% vs 11%).^33^ Open and frank communication between physician and patient about when to cease chemotherapy was recommended, as it was found that patients who received chemotherapy at the end of life had shorter survival compared to those who did not.^31^ Furthermore, there was a non-significant trend for patients to die in their usual residence if they did not receive chemotherapy in the last month of life.^31^

Uncertainty and lack of control resulting from the incurable nature of the disease, lack of clarity around disease progression and when death would occur was perceived to be a primary cause of the emotional and psychosocial distress associated with mesothelioma.^29,32,39^ This was illustrated by descriptions of the diagnosis as a ‘death sentence’^39^ and narratives of existential distress relating to ‘loss of control not only of the body, but also of one’s life’.^32^ A qualitative review identified a theme of hopelessness across three mesothelioma studies due to the incurable nature of the disease and limited prognosis,^29^ whereas other studies noted acceptance of the terminal nature of the illness^34^ and hope for a cure or short/long term survival.^39^ Psychological symptoms described included depression, anxiety and traumatic stress symptoms.^30,37^ Coping strategies employed by people with mesothelioma included: ‘taking life 1 day at a time’^39^; engaging with spiritual or religious rituals and support^39^; focusing on making the best use of time left^34^ and exploring fears and anxieties with a health care professional.^32,35^ Furthermore, some articles described patients employing practical strategies such as putting their financial affairs in order to make the future more predictable, or to reduce the burden on family.^29,34,39^

### Consideration of the impact of seeking compensation

As a disease commonly caused by occupational exposure to asbestos, most people with mesothelioma are eligible for compensation. An Australian survey, that included a compensation scheme as a source of recruitment, found that 97% of participants sought compensation and most participants had learned compensation might be an option from a doctor (62%).^9^ For one patient, who received information about seeking compensation at the time of diagnosis, it was perceived that this was too early as they were overwhelmed by information at that time.^32^ The main reasons described for seeking compensation were to ‘leave their family financially secure’ (67%), for ‘justice’ (59%) and because they ‘needed money to help with treatment costs’ (30%).^9^ As an industrial disease, one study found that patients wanted their doctors to view them as a victim of asbestos,^38^ whereas another described the dilemma of whether to seek compensation from a longstanding employer to whom they felt significant loyalty.^27^ Several qualitative studies described how the additional burden of seeking compensation aggravated an already difficult situation.^27,29,34,35^ The terminal nature of the diagnosis meant that what limited time the patient had left had to be spent meeting lawyers, completing forms and finding relevant documents and evidence of asbestos exposure.^29^ Furthermore, due to the high symptom burden of the disease it was described that seeking compensation had to be a joint effort between the patient and carer, as there are times when the patient was too unwell to pursue the claim.^35^ Moreover, whether or not they would actually receive the compensation was another source of uncertainty.^34^

### Family carer needs

The needs of family carers were described in four of the included papers.^9,17,27,33^ The only randomised controlled trial included in the review identified that routine early referral to specialist palliative care significantly improved carer satisfaction compared to usual care.^17^ Specifically there was increased satisfaction reported with the emotional support provided to family members by the specialist palliative care team, as well as other items related to how the carer perceived care was provided to the patient in terms of: attention to symptoms, management of symptoms, response to symptom changes and emotional support.^17^ A survey identified that 31% of carers would have liked clearer information about malignant pleural mesothelioma^9^ and a qualitative study highlighted the need for information around intimacy as symptoms progressed.^35^ Moreover, family carers would have liked the opportunity to talk to a healthcare professional by themselves,^9,35^ more time with doctors and access to psychological support.^9^ In the studies that included bereaved relatives of people with mesothelioma, concerns were expressed about the lack of bereavement services; bereaved relatives felt they would have benefitted from grief counselling and/or a post-death consultation with a medical or palliative care specialist.^9,27^

## Discussion

This systematic integrative review presents an overview of the palliative care needs of people with mesothelioma and their families. The studies revealed a wide range of palliative care needs including the need for a co-ordinated, team based approach to palliative care, open and frank communication around the curability, prognosis and progression of the disease and the opportunity for patients and carers to explore their fears and anxieties and prepare themselves for the end of life both emotionally and practically. In addition, the process of seeking compensation compounds an already difficult situation by dictating how the limited time the patient and carer have left together is spent. Studies detailing the needs of family carers found they would like the opportunity to speak with a healthcare professional alone and bereaved relatives felt they would have benefitted from grief counselling or a post-death consultation with a doctor.

Evidence from our review suggests people with mesothelioma and their family carers report a wide range of palliative care needs, yet conflicting statements have been reported regarding the benefit of specialist palliative care for these patients (e.g. Brims et al.^17^ and Kao et al.^31^). This may be the result of specialist palliative care services being unable to meet the complex and unique needs of people with mesothelioma. However, perhaps an alternative explanation is that the provision of ‘generalist’ palliative care, by providers already highly skilled in caring for people with mesothelioma, is sufficient to meet the palliative care needs of these patients. Further exploration of the role of thoracic and oncology teams in providing palliative care, and the potential for working in partnership with specialist palliative care, is needed to better understand how to meet the complex needs of this patient group. Indicative of the importance of this, a study exploring healthcare professionals perceptions of caring for people with mesothelioma found 74% of nurses perceived one of their main functions was interacting with palliative care services.^40^

Compared with other cancers, this integrative review confirms some notable differences in the palliative care needs of people with mesothelioma, such as legal and compensation issues, which are almost unique to this patient group and present challenges that health professionals may not be familiar with addressing. A growing evidence base suggests that patients approaching the end of life and their family carers can face considerable financial burden,^41^ and this burden can have negative implications for both patient and carer. For people with mesothelioma, there is a difficult trade-off between financial security and long winded, complex compensation claims. The impact of this on patients and carers should be acknowledged, and the unique circumstances regarding compensation in asbestos related disease should be considered an important element of palliative care for this group.

The cross-cutting theme of uncertainty underpinned the other themes relating to the patients’ needs. Similarly to this study, others have noted that uncertainty at the end of life is often a source of distress, however unlike other populations uncertainty was less commonly discussed in relation to spirituality,^42^ but was primarily related to medical prognosis and when death would occur. As others have proposed, our findings support the importance of healthcare professionals discussing uncertainty with patients and their carers.^43^ Furthermore, uncertainty compounded by lack of co-ordinated care could be alleviated by a co-ordinated team based approach to palliative care with a named point of contact. This reflects the recommendations of the British Thoracic Society, who stress the importance of co-ordinated care facilitated by clinical nurse specialists and recommend a named point of contact in case of need.^14^

The palliative care needs of family carers of people with mesothelioma were only described in five of the included studies. Similarly to the carers of patients with other terminal diagnoses, carers of people with mesothelioma highlighted the need for more care-related information and psychological support.^44^ Tools such as the Carer Support Needs Assessment Tool (CSNAT), which is widely used in palliative care, could be adopted in mesothelioma care as one means of assessing carer needs and directing to appropriate support.^45^ The need for carers of people with mesothelioma to receive one-to-one support from health care professionals, has also been highlighted in a review exploring the psychological needs associated with mesothelioma.^10^ The need for bereavement support, such as grief counselling or a post-death consultation identified in this review,^9,27^ is widely acknowledged but accessing support can be challenging.^46^

### Strengths and limitations

One potential limitation is that we may have overlooked relevant studies; however, we believe this to be unlikely given the rigorous search strategies and manual searching of included papers reference lists. Whilst all of the included articles detail the palliative care needs of people with mesothelioma, the primary focus of many of the articles was not palliative care, but the experience of mesothelioma more broadly, which limited the depth of the findings. Another possible limitation is the heterogeneity of the included studies, which can present a challenge in terms of synthesising findings generated by different methodologies of varying qualities. The integrative review methodology employed in the review enhances the rigour of the synthesis process and ensures relevant information is not overlooked.^21^ The main strength of this review is that it is the first systematically conducted review in this under-researched topic area. The search was comprehensive and the integrative review methodology enabled the synthesis of findings from a wide range of study designs.

### What this review adds

As the first systematic review of the palliative care needs and experiences of people with mesothelioma and their families our findings have important implications for both clinical practice and research. The review demonstrates an overall dearth of evidence for this challenging and rare cancer. Our findings indicate that people with mesothelioma report a range of palliative care needs which need to be understood within the context of their diagnosis. The unique combination of mesothelioma being a rare cancer and an industrial disease means that healthcare professionals need to pay particular attention to the impact the process of seeking compensation has on people with mesothelioma and carers throughout their palliative journey. Understanding these unique circumstances should be central to a co-ordinated, team-based approach to palliative care with a named point of contact.

This review also adds to the broader debate about how to provide palliative care for a growing population of patients with palliative care needs, within a system limited by resources and with specialist palliative care capacity unlikely to see significant expansion. The provision of ‘generalist’ palliative care by a wider range of health and social care professionals has been posed as one solution to this issue, yet many non-specialists feel they lack the requisite skills to provide this care.^47^ Findings from our review support the notion that palliative care needs are often complex and, particularly in a rare cancer like mesothelioma, some generalists lack confidence in addressing these needs. The future development of palliative care services for people with mesothelioma and their carers will require better systematisation of the levels of care provision, as well as exploration of the potential for more partnership working between generalist and specialist palliative care providers. The findings support the need to further understand of the role that clinical nurse specialists play in the delivery of palliative care for rare cancers.^48^ Recent evidence suggests that specialist/advance practice nurses can play an instrumental role in improving the end of life care experience, and are well positioned to address the shortfall of palliative care expertise.^49^ A better understanding of the role of clinical nurse specialists in palliative care for mesothelioma will allow us to draw wider implications for other life limiting conditions.

Understanding of this topic would benefit from an in-depth qualitative exploration focussed specifically on the palliative care needs experienced by people with mesothelioma and their family carers. In addition, further research exploring the quality and content of generalist palliative care currently being provided by healthcare professionals would help to unpick the conflicting findings relating to the benefits of early referral to specialist palliative care.

## Conclusion

This study demonstrates that people with mesothelioma and their carers have a wide range of palliative care needs. The needs of patients and carers were underpinned by a cross-cutting theme of uncertainty and categorised into five areas of need/themes: (1) organisation and co-ordination of services, (2) communication and information needs, (3) management of care needs and high symptom burden, (4) consideration of the impact of seeking compensation and (5) family carer needs. Studies including family carers described the need for one-to-one support for carers from a healthcare professional, access to care-related information and psychological support, as well as bereavement support. The conflicting findings around the importance of early referral to specialist palliative care warrants further investigation into partnership working and the role thoracic and oncology teams play in providing generalist palliative care.

## Supplemental Material

sj-docx-1-pmj-10.1177_02692163211007379 – Supplemental material for Understanding the palliative care needs and experiences of people with mesothelioma and their family carers: An integrative systematic review Click here for additional data file. Supplemental material, sj-docx-1-pmj-10.1177_02692163211007379 for Understanding the palliative care needs and experiences of people with mesothelioma and their family carers: An integrative systematic review by Madeleine Harrison, Clare Gardiner, Bethany Taylor, Stephanie Ejegi-Memeh and Liz Darlison in Palliative Medicine
