# Supplementary material for: Understanding the palliative care needs and experiences of people with mesothelioma and their family carers: An integrative systematic review
Source: Palliat Med. 2021 Apr 8;35(6):1039–51. doi: 10.1177/02692163211007379 (PMC8188997; doi:10.1177/02692163211007379)
Supplement: sj-docx-1-pmj-10.1177_02692163211007379 – Supplemental material for Understanding the palliative care needs and experiences of people with mesothelioma and their family carers: An integrative systematic review [file sj-docx-1-pmj-10.1177_02692163211007379.docx]

**Supplemental appendices**

**Appendix 1**

Search strategies for Medline, PsycINFO, CINAHL and the Cochrane library:

*Medline via Ovid*

1. exp Mesothelioma/

2. mesothelioma.mp. [mp=title, abstract, original title, name of substance word, subject heading word, floating sub-heading word, keyword heading word, organism supplementary concept word, protocol supplementary concept word, rare disease supplementary concept word, unique identifier, synonyms]

3. 1 or 2

4. exp Palliative Care/

5. palliative care.mp.

6. palliative medicine.mp.

7. palliative treatment*.mp.

8. palliative therap*.mp.

9. end of life.mp.

10. end of life care.mp.

11. terminally ill.mp.

12. terminal care.mp.

13. hospice.mp.

14. supportive care.mp.

15. 4 or 5 or 6 or 7 or 8 or 9 or 10 or 11 or 12 or 13 or 14

16. 3 and 15

17. 16 and 2000:2020.(sa_year).

*PsychINFO via Ovid*

1. mesothelioma.mp.

2. exp Palliative Care/

3. palliative care.mp.

4. palliative medicine.mp.

5. palliative treatment*.mp.

6. palliative therap*.mp. [mp=title, abstract, heading word, table of contents, key concepts, original title, tests & measures, mesh]

7. exp "Death and Dying"/

8. end of life.mp.

9. end of life care.mp.

10. exp Terminally Ill Patients/

11. terminally ill.mp. [mp=title, abstract, heading word, table of contents, key concepts, original title, tests & measures, mesh]

12. terminal care.mp.

13. exp Hospice/

14. hospice.mp. [mp=title, abstract, heading word, table of contents, key concepts, original title, tests & measures, mesh]

15. supportive care.mp.

16. 2 or 3 or 4 or 5 or 6 or 7 or 8 or 9 or 10 or 11 or 12 or 13 or 14 or 15

17. 1 and 16

18. 17 and 2000:2020.(sa_year).

*CINAHL via EBSCOhost*

S1 mesothelioma

S2 (MH "Palliative Care")

S3 palliative care

S4 palliative treatment*

S5 palliative medicine

S6 "palliative therap*"

S7 (MH "Hospice and Palliative Nursing")

S8 end of life

S9 end of life care

S10 (MH "Terminally Ill Patients+")

S11 terminally ill

S12 (MH "Terminal Care+")

S13 terminal care

S14 (MH "Hospice Care")

S15 hospice*

S16 supportive care

S17 S2 OR S3 OR S4 OR S5 OR S6 OR S7 OR S8 OR S9 OR S10 OR S11 OR S12 OR S13 OR S14 OR S15 OR S16

S18 S1 AND S17

[filtered search 2000-2020]

*Cochrane library*

#1 mesothelioma 715

#2 palliative care 5550

#3 palliative treatment* 5582

#4 MeSH descriptor: [Palliative Care] explode all trees 1569

#5 end of life 37359

#6 MeSH descriptor: [Terminal Care] explode all trees 438

#7 end of life care 13092

#8 palliative medicine 2423

#9 palliative therap* 6277

#10 MeSH descriptor: [Hospice Care] explode all trees 96

#11 hospice 936

#12 supportive care 8500

#13 terminally ill 386

#14 #2 OR #3 OR #4 OR #5 OR #6 OR #7 OR #8 OR #9 OR #10 OR #11 OR #12 OR #13 50431

#15 #1 and #14 188

Filtering by year (2000-2020)

**Appendix 2**

Results of MMAT for quality appraisal for included primary research

| **Author** | Arber et al (2013) | Bibby et al (2017) | Brims et al (2019) | Dooley et al (2010) | Hughes et al (2008) | Kao et al (2013) | Lee et al (2009) | Mercadante et al (2016) | Clayson et al (2005) | Nagamatsu et al (2014) | Nagamatsu et al (2019) | Walker et al (2019) | Warby et al (2019) |
| --- | --- | --- | --- | --- | --- | --- | --- | --- | --- | --- | --- | --- | --- |
| **Study design** | Qualitative | Quantitative non-randomised | Randomised controlled trial | Quantitative descriptive | Qualitative | Quantitative descriptive | Qualitative | Quantitative descriptive | Qualitative | Quantitative non-randomised | Quantitative descriptive | Qualitative | Quantitative descriptive |
| **Q1** | Y | Y | Y | N | Y | Y | Y | Y | Y | C | Y | Y | Y |
| **Q2** | Y | Y | Y | N | Y | Y | Y | Y | C | Y | N | Y | Y |
| **Q3** | Y | Y | Y | Y | Y | Y | Y | Y | Y | Y | Y | Y | Y |
| **Q4** | Y | Y | N | C | Y | N | Y | Y | Y | N | N | Y | N |
| **Q5** | Y | Y | Y | Y | Y | Y | Y | Y | Y | Y | y | Y | Y |

*Key: yes = Y, no = N, can’t tell = C*

**Appendix 3**

Results of JBI review checklist for quality appraisal of included reviews

|  | **Questions** | **Ball et al (2016)** |
| --- | --- | --- |
| **1** | Is the review question clearly and explicitly stated? | Yes |
| **2** | Were the inclusion criteria appropriate for the review question? | Yes |
| **3** | Was the search strategy appropriate? | No |
| **4** | Were the sources and resources used to search for studies adequate? | Yes |
| **5** | Were the criteria for appraising studies appropriate? | Yes |
| **6** | Was critical appraisal conducted by two or more reviewers independently? | Unclear |
| **7** | Were there methods to minimize errors in data extraction? | No |
| 8 | Were the methods used to combine studies appropriate? | Yes |
| 9 | Was the likelihood of publication bias assessed? | N/A |
| 10 | Were recommendations for policy and/or practice supported by the reported data? | Yes |
| 11 | Were the specific directives for new research appropriate? | Yes |
